# Prevalence of heavy menstrual bleeding, iron deficiency, iron deficiency anemia, and treatment in women with von Willebrand disease—a cohort study

**DOI:** 10.1016/j.rpth.2025.102949

**Published:** 2025-06-20

**Authors:** Linda Myrin–Westesson, Petra Elfvinge, Eva Zetterberg, Anna Olsson

**Affiliations:** 1 Department of Medicine, Sahlgrenska University Hospital, Region Västra Götaland, Gothenburg, Sweden; 2 Institution for Nursing Science and Health, Sahlgrenska Academy, University of Gothenburg, Gothenburg, Sweden; 3 Coagulation Center, Karolinska University Hospital, Karolinska Institutet, Stockholm, Sweden; 4 Department of Hematology, Oncology and Medical Physics, Skåne University Hospital, Region Skåne, Malmö, Sweden; 5 Department of Internal Medicine, Sahlgrenska Academy, University of Gothenburg, Gothenburg, Sweden

**Keywords:** anemia, blood coagulation disorders, iron deficiencies, menorrhagia, von Willebrand disease

## Abstract

**Background:**

A major challenge for women with von Willebrand disease (VWD) is heavy menstrual bleeding (HMB). HMB is linked to iron deficiency (ID) and ID anemia, both of which are related to morbidity that can impair cognitive and physical health.

**Objectives:**

The aim of the study was to evaluate the prevalence of HMB, ID, ID anemia, the treatments used, and the impact of HMB among women aged 18 to 55 years with VWD.

**Methods:**

A cross-sectional nationwide study was conducted in Sweden. Women aged 18 to 55 years with VWD were recruited from the National Registry. Data collection included questionnaires assessing HMB, prevalence, treatment, and its impact on life. Hemoglobin and ferritin levels were collected, and clinical data were retrieved from medical records.

**Results:**

A total of 208 women were identified, and 136 (66%) participated in the study. Data revealed that 45% of the women had low ferritin levels (<30 μg/L), and 18% had hemoglobin levels < 12.0 g/dL. Tranexamic acid was used by 64% of the women, hormone therapy by 37%, von Willebrand factor concentrate by 18%, and desmopressin by 8%. HMB was reported by 66% of the women; HMB significantly impacted life, particularly work/school, physical activity, and intimacy.

**Conclusion:**

The high prevalence of ID highlights the importance of regular follow-up and proactive iron management. The results indicate that more women with type 1 and 2 VWD may require von Willebrand factor concentrate, despite it generally being considered a last-line treatment. The loss to follow-up observed in a substantial proportion of women underscores the need for improved long-term care strategies to prevent complications.

## Essentials

• HMB is a major challenge for women with von Willebrand disease.
• This nationwide data collection study included questionnaires assessing HMB and ferritin levels.
• Among menstruating women, HMB was reported by 66%, and 62% had low ferritin levels.
• The results highlight the importance of regular follow-up and proactive iron management.

## 1 Introduction

von Willebrand disease (VWD) is the most common inherited bleeding disorder, clinically affecting approximately 1 in 1000 persons, with an equal incidence among males and females [1]. VWD is classified into 3 subtypes. Type 1, the most common, is characterized by partial deficiency of von Willebrand factor (VWF) activity. Type 2 is classified into 2A, 2B, 2M, and 2N based on functional VWF activity differences and is characterized by qualitative defects. VWD type 3 is characterized by virtually complete deficiency of VWF activity [2]. Bleeding severity typically increases from type 1 to type 3, but there is variation in bleeding phenotype among all types of the disease [3].

VWD is characterized by mucocutaneous bleeding and bleeding following surgical procedures and trauma. In the most severe forms of the disorder, muscle hematomas and joint bleeds may also occur [4]. The clinical presentation is often more pronounced in females due to additional hemostatic challenges related to menstruation and childbirth. Heavy menstrual bleeding (HMB) constitutes a common and significant manifestation of VWD, with a prevalence exceeding 50% to 80% among affected women, as reported in several studies [[5], [6], [7]].

In clinical practice, HMB is defined as excessive menstrual bleeding that negatively impacts a woman’s physical, social, emotional, and material quality of life [8,9]. In terms of blood loss, HMB is defined as menstrual bleeding exceeding 80 mL per cycle [10]. HMB is a significant factor contributing to lower health-related quality of life scores in females with VWD [[11], [12], [13]]. Women with VWD and HMB are at an increased risk of developing iron deficiency (ID) and ID anemia (IDA), both of which are linked to morbidity that can impair cognitive and physical health, including the ability to participate fully in school and work [[14], [15], [16]]. ID with a ferritin level of < 30 μg/L indicates a significant depletion of iron stores in the body. Ferritin at this level is typically associated with an increased risk of developing IDA [17,18].

The most common treatment for HMB includes hormonal therapies, such as oral contraceptives, progestin, or intrauterine devices to regulate menstrual cycles and reduce bleeding. In cases where hormonal treatments are ineffective or not well-tolerated, options such as tranexamic acid (TXA) or surgical interventions may be considered. Among women with VWD, conventional treatments for HMB may be inadequate due to the underlying hemostatic defect. In women with VWD, desmopressin and VWF concentrate (VWFC) may be necessary to manage HMB, as these therapies address the underlying hemostatic defect. Desmopressin is particularly effective in type 1 and some type 2 VWD, while VWFC is required in more severe cases to ensure proper hemostasis during menstruation. The VWFC treatment option is both time-consuming and demanding, as it involves intravenous injections [3,19,20].

HMB is a significant issue for women with VWD, highlighting the need for further understanding. Additionally, more knowledge is needed regarding the treatments offered to patients and their effectiveness in managing HMB. The aim of this study, therefore, was to evaluate the prevalence of HMB, ID, and IDA and the treatments used to manage HMB among women aged 18 to 55 years with VWD in Sweden, as well as to examine the impact of HMB on personal, professional, and social life within the same cohort.

## 2 Methods

### 2.1 Study design and participants

A cross-sectional, nationwide, multicenter study was conducted at the 3 existing European Hemophilia Comprehensive Care Centers (EHCCCs) in Sweden. The study followed Strengthening the Reporting of Observational studies in Epidemiology (STROBE) checklist throughout the research process [21]. Participants were included between December 2023 and December 2024. Study information, the informed consent form, and the study-specific questionnaire were sent by post. Duplicates were sent to women who had not responded within a month. Written informed consent was obtained from the participating women, and the study was approved by the Swedish Ethical Review Authority (2023-06262-01).

Women aged 18 to 55 years with type 1 VWD with VWF activity ≤ 0.35 IU/mL, type 2 VWD, and type 3 VWD were eligible for recruitment. In the Swedish National Registry of bleeding disorders, a diagnosis of VWD type 1 is defined when VWF activity is ≤ 0.35 IU/mL, in accordance with the current Nordic Hemophilia Council guidelines [22]. A total of 208 women were identified in the Swedish National Registry of bleeding disorders [23].

### 2.2 Data collection

A questionnaire developed for the study was used for the collection of data on the prevalence of self-reported HMB and the treatments provided. The questionnaire also included the patient-centered definition of HMB presented by the *VWD guidelines panel* in 2021 [24]. HMB is defined as menstrual bleeding lasting ≥8 days associated with repeated passing of blood clots, soaking through ≥1 pads/tampons every 2 hours on multiple days, requiring use of >1 pad/tampon at a time, needing to change a pad/tampon overnight, or a Pictorial Blood Loss Assessment Chart (PBAC) score of >100 [24]. Participants were instructed to complete 2 PBACs during 2 consecutive menstrual cycles. The PBAC is a validated tool used to quantify menstrual blood loss. Participants record the degree of soiling on sanitary products, as well as the number of pads and tampons used during each cycle. Clots and instances of flooding were also documented. Each entry is assigned a score, allowing an objective assessment of blood loss. A PBAC score exceeding 100 is used to define HMB [25,26]. This method provides a standardized approach to evaluating menstrual blood loss in clinical and research settings [27]. The study-specific questionnaire also included questions about how HMB impacts personal and social life in terms of absence from school and work, physical activity, social activity, and intimacy.

After inclusion in the study, blood samples were obtained either at local hospitals or at the EHCCCs, based on the participants’ choice. The blood tests included measurements of hemoglobin and ferritin. Anemia in nonpregnant women was defined as hemoglobin level < 12.0 g/dL, according to the World Health Organization [28]. Ferritin levels < 30 μg/L were used for ID diagnosis in the present study [29,30].

Clinical data were collected from the Swedish National Registry for bleeding disorders and medical records by healthcare providers (HCPs) at the 3 EHCCCs. This included type and severity of VWD, baseline factor levels, and age of diagnosis. Updated samples were obtained for participants whose factor level measurements were >10 years old. All baseline samples were collected after participants reached the age of 17.

### 2.3 Statistical analysis

Data were analyzed using SPSS software (version 28.0, IBM, SPSS). Descriptive data were presented as numbers and percentages, and median and range. The Pearson chi-squared test was used to compare groups by assessing whether there was significant association between categorical variables, while the *t*-test was employed to compare differences between continuous variables. A *P* value < .05 was considered statistically significant throughout the results.

## 3 Results

A total of 208 eligible women aged 18 to 55 years with VWD were identified in the Swedish National Registry for bleeding disorders and invited to participate. A total of 136 women (66%) participated in the study. The nonrespondent group (*n* = 72) had a median age of 36 years (range, 18-54); 53% had type 1 VWD, 22% had type 2A, 4% had type 2B, 17% had type 2M, and 4% had type 3 VWD.

### 3.1 Clinical characteristics of participants

The median age of the participants was 36 years (range, 18-55), and median age at diagnosis was 11 years (range, 1-46). The majority of participants had type 1 VWD (35%, *n* = 48), 32% (*n* = 44) had type 2A, 7% (*n* = 10) had type 2B, 14% (*n* = 19) had type 2M, 4% (*n* = 5) had type 2N, and 7% (*n* = 10) had type 3 VWD (Table 1).

**Table 1:** Clinical characteristics of participants.

Characteristics	All, *N* = 136, median (range)	Type 1, *n* = 48, median (range)	Type 2A, *n* = 44, median (range)	Type 2B, *n* = 10, median (range)	Type 2M, *n* = 19, median (range)	Type 2N, *n* = 5, median (range)	Type 3, *n* = 10, median (range)
Age (y)	36.0 (18-55)	37.5 (19-53)	36.5 (18-55)	36.0 (19-54)	34.0 (20-53)	42.0 (25-47)	35.5 (19-51)
Age when diagnosed (y)	11.0 (1-46)	19.5 (1-46)	8.0 (1-45)	10.5 (4-29)	16.0 (1-42)	29.0 (10-37)	2.5 (1-10)
VWF activity IU/mL	0.16 (<0.04-1.01)	0.24 (0.04-0.35)	0.14 (0.04-0.28)	0.18 (0.08-0.32)	0.15 (0.05-0.29)	0.62 (0.29-1.01)	0.05 (<0.04-0.08)
VWF antigen IU/mL^a^	0.23 (<0.04-0.85)	0.25 (0.07-0.41)	0.22 (0.10-0.80)	0.44 (0.23-0.62)	0.23 (0.12-0.74)	0.42 (0.29-0.85)	0.04 (<0.04-0.10)
FVIII:C IU/mL	0.42 (0.01-1.18)	0.52 (0.06-1.18)	0.35 (0.16-0.86)	0.58 (0.32-0.68)	0.53 (0.23-1.00)	0.26 (0.14-0.40)	0.04 (0.01-0.14)

	**% (*n*)**	**% (*n*)**	**% (*n*)**	**% (*n*)**	**% (*n*)**	**% (*n*)**	**% (*n*)**
Menstruation in past y	54.4 (74)	45.8 (22)	54.5 (24)	60.0 (6)	73.7 (14)	80.0 (4)	40.0 (4)
No menstruation due to:							
Hormonal therapy/IUD	72.6 (45)	73.1 (19)	70.0 (14)	25.0 (1)	100.0 (5)	100.0 (1)	83.3 (5)
Hysterectomy	8.1 (5)	11.6 (3)	5.0 (1)	25.0 (1)	−	−	−
Menopause	9.7 (6)	7.7 (2)	10.0 (2)	25.0 (1)	−	−	16.7 (1)
Pregnancy	6.4 (4)	7.7 (2)	10.0 (2)	−	−	−	−
Breastfeeding	3.2 (2)	−	5.0 (1)	25.0 (1)	−	−	−

FVIII:C, factor VIII activity; IU, International Units; IUD, intrauterine device; VWF, von Willebrand factor.

a Data not available for 6 participants.

The prevalence of menstruation in the past year varied across age groups. Among participants aged 18 to 30 years, 57% reported having menstruated in the past year, while the prevalence was higher among participants aged 31 to 40 years, where 78% reported menstruation in the past year. In contrast, only 35% of those aged >41 reported having menstruated in the past year. Most of the nonmenstruating women had secondary amenorrhea due to hormonal treatment or hysterectomy. The remaining participants had either reached menopause, had undergone a hysterectomy, or were pregnant/breastfeeding (Table 1).

Approximately one-third (37%) of the menstruating women underwent hormonal treatment for HMB. The majority of participants used TXA to prevent HMB, while a smaller proportion of the women who had menstruated in the past year used desmopressin or VWFC to prevent or treat it.

Overall, 24% of the cohort reported bleeding other than HMB in the past year. The most common types of additional bleeding were epistaxis, followed by bleeding related to gynecological surgery, minor surgery, and postpartum hemorrhage. In the cohort, 73% had attended at least 1 follow-up visit at EHCCCs in the past 5 years. Among women who had menstruated in the past year, 76% had a follow-up visit within the same period.

### 3.2 HMB

Self-reported HMB was observed in 66% of participants who had menstruated in the past year. In the age groups 18 to 30 and 31 to 40 years, 71% reported HMB. A lower prevalence was recorded among participants >41 years of age, with 53% reporting HMB. In the total cohort (excluding women who were menopausal, had undergone a hysterectomy, or were pregnant or breastfeeding), a significantly higher proportion of women not using hormonal therapy reported HMB compared with those using hormonal therapy (*P* < .001). Of the women using hormonal therapy, 90% reported that the treatment was effective against HMB.

Excluding hormone therapy, 69% of the women reported using medication to manage menstrual bleeding. The use of TXA was reported by 64% of participants. Additionally, 8% reported using desmopressin, and 18% required factor concentrate as part of their treatment regimen. Among women using factor concentrate for the prevention or treatment of HMB, 9% had type 1, 19% had type 2, and 50% had type 3. TXA was utilized for HMB treatment across all VWD types.

In the total cohort, 13% (*n* = 17) of the women were using iron substitution. Of the women who had menstruated in the past year, 12% (*n* = 9) were using iron substitution (Table 2).

**Table 2:** Medication used by women who had menstruated in past year, reported by participants.

Medication	All types, *N* = 74% (*n*)	Type 1, *n* = 22% (*n*)	Type 2, *n* = 48% (*n*)	Type 3, *n* = 4% (*n*)
Self-reported HMB	66.2 (49)	68.2 (15)	68.8 (33)	25.0 (1)
Use of hormonal therapy	36.5 (27)	22.7 (5)	37.5 (18)	100.0 (4)
Effective	81.5 (22)	80.0 (4)	77.8 (14)	100.0 (4)
Medication to reduce menstruation (excluding hormone therapy)	68.9 (51)	95.5 (16)	66.7 (32)	75.0 (3)
Use of TXA	63.5 (47)	72.7 (16)	62.5 (30)	25.0 (1)
Effective	72.3 (34)	81.3 (13)	66.7 (20)	100.0 (1)
Use of desmopressin^a^	8.1 (6)	13.6 (3)	6.3 (3)	−
Effective	66.7 (4)	66.7 (2)	66.7 (2)	−
Use of VWFC	17.6 (13)	9.1 (2)	18.8 (9)	50.0 (2)
Effective	92.3 (12)	100.0 (2)	88.9 (8)	100.0 (2)
Use of iron therapy^b^	12.2 (9)	−	18.7 (9)	−

HMB, heavy menstrual bleeding; TXA, tranexamic acid; VWF, von Willebrand factor; VWFC, VWF concentrate.

a Nasal desmopressin was not available from 2021 onward.

b Currently using an iron substitution.

Of the women who menstruated in the past year, 19 (26%) reported that their menstruation lasted 8 days or longer. Thirty-nine women (53%) reported consistently soaking through ≥1 sanitary products every 2 hours on multiple days. Twenty-seven women (37%) required the use of >1 sanitary product at a time. Thirty-eight women (47%) needed to change their sanitary protection during the night, and 53 women (72%) reported repeated passing of blood clots.

All the women who self-reported HMB also met the patient-centered definition of HMB presented by the *VWD guidelines panel*. Overall, 70 women (95%) who menstruated had HMB, as defined by these guidelines [23].

### 3.3 PBACs

Among the participants who had menstruated in the past year and completed the PBAC, the majority had a score indicative of HMB on the PBAC instrument. A total of 94 PBAC scores were collected from 47 women, with 66% reporting a score of ≥100. In the 18 to 30-year age group, 87% of the women had a PBAC score of ≥100. In the 31 to 40-year age group, 62% had a score of ≥100, while in the oldest age group (>41 years), 55% had a score of ≥100. The median PBAC score in the entire cohort was 148 points, with a wide range of 5 to 673 points. Moreover, 93% of the women who reported HMB had a PBAC score of ≥100.

Among women with type 1 VWD, 73% reported a PBAC score of ≥100 compared with 71% of those with type 2 VWD. In contrast, only 25% of women with type 3 VWD reported a high PBAC score. The median PBAC score was 192 for women with type 1 VWD, 132 for those with type 2 VWD, and 50 for those with type 3 VWD. The highest recorded score among women with type 3 VWD was 148, while both type 1 and type 2 VWD groups included individuals with scores exceeding 600.

### 3.4 Ferritin and hemoglobin

The distribution of ferritin and hemoglobin levels is presented in Figure, stratified by women who had menstruation in the past year vs those who did not, and by those who used hormone treatment vs those who did not.

**Figure:**
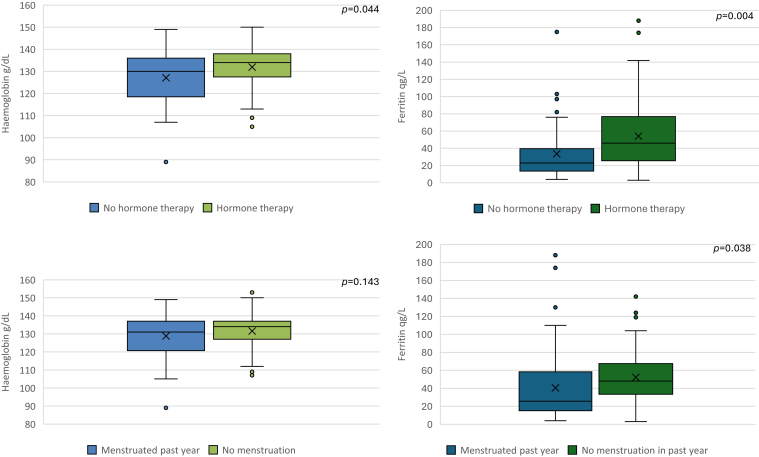
Measurements of hemoglobin and ferritin levels stratified by women who had menstruation in the past year vs those who did not, and by those who used hormone treatment vs those who did not. The boxes represent median values with upper and lower quartiles, and the dots represent outliers. The “X” represents the mean value for each group. Statistical analyses were based on comparisons between groups. *P* values were generated using a *t*-test.

Overall, 18% of the women in the entire cohort had hemoglobin levels < 12.0 g/dL (Table 3). No significant differences in hemoglobin levels were observed between women who had menstruated in the past year and those who had not (*P* = .23). Hemoglobin levels < 12.0 g/dL were observed in all subtypes of VWD except for type 2N.

**Table 3:** Ferritin and hemoglobin levels.

Parameters	All types, *N* = 136% (*n*)	Type 1, *n* = 48% (*n*)	Type 2, *n* = 78% (*n*)	Type 3, *n* = 10% (*n*)
**Total cohort**				
Ferritin 0-15 μg/L	19.5 (25)	6.8 (3)	26.7 (20)	20.0 (2)
Ferritin 0-29 μg/L	45.3 (58)	34.1 (15)	49.3 (37)	40.0 (4)
Ferritin > 30 μg/L^a^	54.7 (70)	65.9 (29)	50.7 (38)	40.0 (4)
Hemoglobin < 12.0 g/dL^b^	18.0 (24)	10.9 (5)	22.7 (17)	20.0 (2)
**Menstruation in the past y**	**54.4 (74)**	**45.8 (22)**	**61.5 (48)**	**40.0 (4)**
Ferritin 0-15 μg/L	25.7 (19)	13.6 (3)	29.2 (14)	50.0 (2)
Ferritin 0-29 μg/L	62.2 (46)	59.1 (13)	60.4 (29)	100.0 (4)
Ferritin > 30 μg/L	37.8 (28)	40.9 (9)	39.6 (19)	−
Hemoglobin < 12.0 g/dL	21.6 (16)	13.6 (3)	25.0 (12)	25.0 (1)
**No menstruation in the past y**	**72.6 (62)**	**54.2 (26)**	**38.5 (30)**	**60.0 (6)**
Ferritin 0-15 μg/L	11.1 (6)	−	22.2 (6)	−
Ferritin 0-29 μg/L	22.2 (12)	9.1 (2)	29.6 (8)	33.3 (2)
Ferritin > 30 μg/L^a^	77.8 (42)	90.9 (20)	70.7 (19)	66.6 (4)
Hemoglobin < 12.0 g/dL^b^	13.6 (8)	8.3 (2)	17.2 (5)	−

a Data not available for 7 participants.

b Data not available for 3 participants.

Among women who had menstruated in the past year, hemoglobin levels < 12 g/dL were observed in 14% of those with type 1 VWD, 25% with type 2A, 17% with type 2B, 36% with type 2M, and 25% with type 3 VWD.

A significant difference in hemoglobin levels was observed between women who reported HMB and those who did not. Of those reporting HMB, 31% had hemoglobin levels < 12.0 g/dL, while among those not reporting HMB, only 4% had levels < 12.0 g/dL (*P* = .009). In the group who had menstruated in the past year with hemoglobin levels < 12.0 g/dL, approximately one-fifth (19%) reported the occurrence of bleeding other than menstruation in the past year. These included 1 episode of nose bleeding, 2 minor surgeries, and 1 postpartum bleeding among 3 women.

Low ferritin levels (< 30 μg/L) were observed in 45% of the women, and ferritin levels < 15 μg/L were observed in 20% of the total cohort of participants (Table 3). A significant difference was observed between women who had menstruated in the past year and those who had not. Among the women who had menstruated, 62% had ferritin levels < 30 μg/L, while 22% of those who had not menstruated exhibited ferritin levels < 30 μg/L (*P* < .001).

Among the women who had menstruated in the past year, all participants with type 3 VWD had ferritin levels < 30 μg/L. More than half of the women with type 1 or 2 VWD and who had menstruated in the past year had ferritin levels < 30 μg/L (59% with type 1 VWD, 67% with type 2A, 50% with type 2B, 57% with type 2M, and 50% with type 2N). In the group who had menstruated in the past year with ferritin levels < 30 μg/L, approximately a quarter (24%) reported the occurrence of bleeding other than menstruation in the past year. These were single episodes of nose bleeding, minor surgeries, and postpartum bleeding in 11 women. The majority (63%) of women who were using an iron substitution had ferritin levels < 30 μg/L. Almost one-third (29%) of the women who were using iron substitution had hemoglobin levels < 12.0 g/dL.

Regarding low ferritin levels < 30 μg/L, there was a significant difference between women who used hormone therapy and those who did not (*P* = .002), excluding women who were menopausal, had undergone a hysterectomy, or were pregnant or breastfeeding. However, regarding low hemoglobin levels, no significant difference was seen between the women who used hormone therapy and those who did not (*P* = .089). Among women who had menstruated in the past year, no significant difference in ferritin levels < 30 μg/L was observed between those who reported HMB and those who did not (*P* = .78). Additionally, regarding hemoglobin < 12.0 g/dL or ferritin levels < 30 μg/L, no significant differences were found between women with a PBAC score ≥100 and those with a lower PBAC score (*P* = .645 and *P* = .55, respectively).

### 3.5 Impact of HMB on personal and social life

Of the women who had menstruated in the past year, 55% stated that their menstruation generally impacted their lives. No significant differences were seen between age groups of 18 to 30 years, 31 to 40 years, and 41 to 55 years (*P* = .957). The most impacted areas were physical activity (63%) and school or work performance (61%). The women also reported that menstruation impacted intimacy (56%) and social activities (44%). Additionally, the women described that menstruation in the past year negatively impacted their psychological health and sleep.

The participants who reported HMB (*n* = 49, 66%) generally experienced a greater impact of menstruation on their lives than those who did not report HMB (*P* = .001). The difference between women who reported HMB and those who did not was significant across all 4 questions. Women reporting HMB were more likely to report that their menstruation impacted school or work performance (*P* = .005), physical activity (*P* = .001), social activity (*P* = .019), and intimacy (*P* = .01) than women without HMB. Among the women who self-reported HMB, no significant differences were seen between the age groups for any of the 4 questions.

Participants with a PBAC score of ≥100 (72%) were significantly more likely to report a general impact of menstruation on their lives than those with a score of <100 (*P* = .003). Women with scores of ≥100 also reported a greater impact on their school or work performance (*P* = .026) and physical activity (*P* = .026) than those with lower scores. However, no significant differences were observed in the domains of social activity (*P* = .42) or intimacy (*P* = .23).

## 4 Discussion

This nationwide study on women with VWD demonstrates that almost half of the women had ID. Two-thirds of participants who menstruated reported HMB, with the highest prevalence observed in the 18 to 30-year age group. There was a high prevalence of HMB, despite the women being treated and monitored at an EHCCC. This high prevalence highlights that HMB remains a pervasive issue among women with VWD. The results also demonstrate that HMB has a significant impact on their daily lives.

In the present study, the patient-centered definition of HMB (including PBAC) proposed by the *VWD guidelines panel* was used to evaluate the bleeding pattern in a standardized manner [24]. The results revealed a prevalence of HMB in 95% of the cohort of menstruating women, including a proportion of women who did not evaluate and report the bleeding as excessive themselves. As a result, women who reported having normal menstruation were classified as having HMB [24], which may indicate an overestimation of HMB if this particular definition is applied.

The fact that PBAC was completed by only 64% of menstruating women is likely due to the complexity of completing it, often retrospectively at the end of the day, which may have contributed to reluctance on the part of some participants to do so. This emphasizes the challenge of implementing and interpreting PBAC in clinical practice. Of further note is that PBAC scoring used in the present study has not been validated for modern sanitary products, which have a higher absorbency, suggesting that PBAC scores in this study have not been overestimated. This underscores the problems of evaluating HMB and actual blood loss, highlighting the importance of follow-up and generous blood sampling to diagnose or rule out ID and IDA.

Data revealed that 45% of the women had low ferritin levels (< 30 μg/L), and 18% had hemoglobin levels < 12.0 g/dL. The significant difference in ferritin levels between menstruating and nonmenstruating women demonstrates the direct impact of menstrual bleeding on iron stores. The same or higher prevalence of ID and IDA has been reported in previous studies of women with low VWF levels and VWD [31,32].

The most reliable indicator of ID is ferritin, and the optimal cutoff for diagnosis has been the subject of ongoing discussion. Ferritin levels < 15 μg/L are indicative of ID. However, using a cutoff of 30 μg/L improves sensitivity while maintaining specificity in diagnosing ID [29]. A key consideration is that this cutoff helps identify low ferritin levels before more severe ID and IDA develop, allowing for timely initiation of treatment and thereby improving health outcomes. Despite the fact that 62% of the women who had menstruated in the past year had low ferritin levels, only 12% were receiving ongoing iron treatment. This further emphasizes the importance of regular visits and generous blood sampling for ferritin and hemoglobin levels.

In the present study, 27% of the women were lost to follow-up, defined as >5 years since their last visit (including phone or digital visits) to an EHCCC. A recent study conducted in Canada reported similar rates of loss to follow-up in the patient group [33]. Lack of follow-up increases the risk of developing ID and IDA, along with associated complications. HMB leading to ID and IDA occurs in women of reproductive age, even in the absence of an underlying bleeding disorder. The prevalence of ID and IDA varies, depending on factors such as access to healthcare, socioeconomic status, dietary habits, and history of multiple pregnancies [34,35]. However, in present study, only women who are monitored at an EHCCC were included, making the high prevalence of ID and IDA particularly noteworthy.

There are a limited number of studies on the efficacy of various treatment options for HMB for women with VWD, and international VWD guidelines indicate low certainty regarding the effectiveness of therapies [3,36,37]. Since hormonal therapy has been shown to be effective in controlling HMB, based on data from women without bleeding disorders, it is considered the most effective approach, followed by TXA and desmopressin [38]. Although VWFC is safe to use for reducing HMB, it is generally considered a last-line treatment, with the exception of severe forms of VWD [39]. The results of the present study also indicate that women with type 1 and 2 VWD may need VWFC treatment for HMB, as stated in the international guidelines [3]. The results are in line with findings from a recently conducted international prospective study, which demonstrated the effect of VWFC for treatment of HMB in women with VWD [40].

Moreover, the treatment regime with VWFC for general bleeding may not be directly applicable to HMB, as menstruation is physiological bleeding for women. Patients and HCPs cannot rely on a treatment option without close monitoring, particularly when it comes to dosage adjustments and the combination of treatment options, including VWFC. Close collaboration with a gynecologist is often necessary for a successful outcome [31]. A practical requirement for treatment with VWFC is that the woman can administer the treatment at home. Home treatment requires learning and takes time for both the patient and HCP, which can sometimes be a barrier to optimal treatment.

The significant impact of HMB on the personal, professional, and social lives of women with VWD is not unexpected, as this finding aligns with previous studies [12,41,42]. In the present study, more than half of the women who had menstruated in the past year reported that their menstruation generally affected their daily lives, with the greatest impact observed on physical activity and work performance. More proactive management of HMB could reduce this burden, enabling women with VWD to participate in physical activity and work performance on equal terms, thereby fostering both physical and mental well-being.

Further research is needed to better understand the long-term outcomes of different treatment strategies for HMB in women with VWD. Additionally, studies on the psychosocial impact of HMB and effective strategies to support women with VWD in managing the associated challenges are warranted. Strengths of this study include the use of a nationwide population cohort, the collection of ferritin and hemoglobin levels, and the assessment of HMB using 2 different methods. An inclusion rate of 66% was achieved. This may have introduced bias, potentially leading to an overestimation of the prevalence of certain outcomes, and should be taken into account when interpreting the results. Furthermore, the small sample sizes for certain VWD subtypes (eg, types 2B, 2N, and 3) may limit the generalizability of the findings. Additionally, not all menstruating participants completed the PBAC, which could introduce bias. Data on race/ethnicity were not collected, which is a limitation regarding the assessment of socio-cultural determinants of health. Regarding treatment with desmopressin, the nasal spray has not been available on the market since 2020, which has affected the number of women using the medication. The distribution of VWD subtypes differed slightly between responders and nonresponders, with a higher proportion of type 1 VWD in the nonrespondent group and a higher proportion of type 2A VWD among participants, suggesting potential selection bias.

## 5 Conclusion

This nationwide study demonstrates that HMB remains a significant and prevalent issue among women with VWD, despite specialized care at EHCCCs. The high prevalence of ID and IDA highlights the importance of regular follow-up and proactive iron management. Our findings also emphasize the challenges of diagnosing and assessing HMB, particularly with PBAC. Treatment strategies for HMB in women with VWD require individualized approaches, often necessitating a combination of therapies and close collaboration between EHCCCs and gynecologists. While hormonal therapy remains the primary treatment option, our results indicate that more women with type 1 and 2 VWD may require VWFC, despite it generally being considered a last-line treatment. The loss to follow-up observed in a substantial proportion of women underscores the need for improved long-term care strategies to prevent complications such as ID and IDA.
